# Robotic major hepatectomy and laparoscopic major hepatectomy: is there no difference in the rate of intraoperative blood transfusion?

**DOI:** 10.1097/JS9.0000000000001600

**Published:** 2024-05-09

**Authors:** Na Wang, Dan-Na Xie, Bao-Lin Qian

**Affiliations:** a Department of Oncology, Zigong First People’s Hospital, Zigong; b The First Clinical Medical College of Lanzhou University; c Department of Hepatobiliary Surgery, Affiliated Hospital of Southwest Medical University, Luzhou, People’s Republic of China

*Dear Editor,*

We read with great interest the recent article by Mao *et al*.^1^ titled ‘Comparison of safety and effectiveness between robotic and laparoscopic major hepatectomy: a systematic review and meta-analysis’ published in the *International Journal of Surgery*. Hepatectomy is one of the most common surgical treatments for hepatic tumors; how to improve its effectiveness and safety is a serious concern for surgeons. Traditional laparoscopic liver resection meets these requirements to some extent, but newer robotic hepatectomy in the last decade seems to offer a better approach. The study by Mao *et al*. compared the safety and effectiveness between robotic major hepatectomy (RMH) and laparoscopic major hepatectomy (LMH), and found that no significant differences in mortality (OR=1.23, 95% CI=0.50–2.98, *P*=0.65), overall postoperative complications (OR=0.83, 95% CI=0.65–1.06, *P*=0.14), operative time (MD=6.47, 95% CI= −14.72 to 27.65, *P*=0.55), and readmission (OR=0.63, 95% CI=0.28–1.44, *P*=0.27) between RMH and LMH. This study provides vital insights into guidance on clinical decisions in a rapidly evolving field^1^, for which we congratulate the authors on this well-conducted review and meta-analysis.

We note that the authors found that the intraoperative blood loss of the RMH group was significantly less than that of the LMH group (MD= −91.42, 95% CI= −142.18 to −40.66, *P*=0.0004). RMH can reach areas and angles that traditional laparoscopic instruments cannot reach, especially in narrow anatomical areas, which may be an important reason for the reduction of intraoperative bleeding^2^. Interestingly, the authors found that there was no significant difference in intraoperative blood transfusion between RMH and LMH (OR=0.77, 95% CI=0.55–1.08, *P*=0.13), which seems to be unreasonable. Although RMH did not show a benefit over LMH in intraoperative blood transfusion, there was moderate heterogeneity in the included studies (*I* ^2^=41%). In general, it represents moderate heterogeneity when *I* ^2^ ranges from 30% to 70%^3^, and we agree with the authors to use random effects model for meta-analysis when *I* ^2^ ≤50%.

Considering the impact of heterogeneity on the results, we conducted ‘the One Study Removed meta-analysis’ for the included studies. Comprehensive Meta-Analysis (Version 3, Biostat, Englewood, New Jersey, USA) was used for analysis as previously reported^4^. We found that the rate of intraoperative blood transfusion showed significant changes between RMH and LMH (*P*=0.044), which was only discovered after the removal of the Chong (2022) study (Fig. 1).

**Figure 1:**
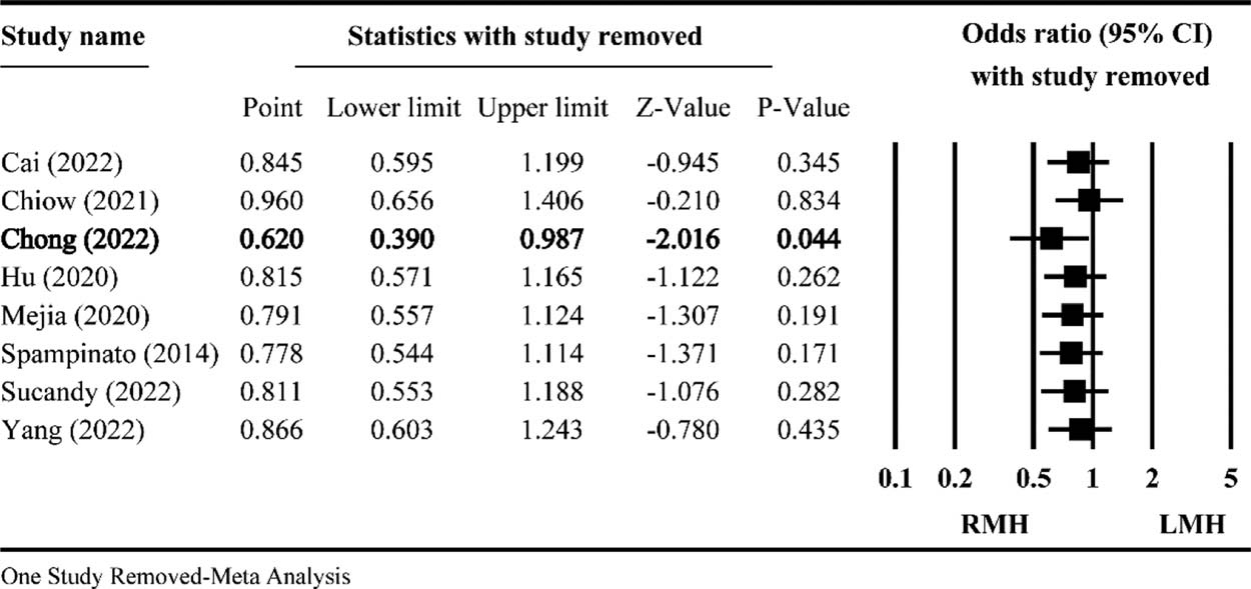
The One Study Removed meta-analysis. LMH, laparoscopic major hepatectomy; RMH, robotic major hepatectomy.

In summary, the well-executed meta-analysis by Mao *et al*.^1^ offers evidence to support the beneficial role of RMH in liver resection. Our analysis confirmed that RMH reduces the risk of intraoperative blood transfusion in patients compared to LMH. However, further randomized studies are still needed to investigate whether RMH has an advantage over LMH.

## Ethical approval

Not applicable.

## Consent

Not applicable.

## Sources of funding

No external funding was received for this study.

## Author contribution

N.W. and D.-N.X.: data curation, formal analysis, methodology, software, validation, writing – original draft, and writing – review and editing; B.-L.Q.: conceptualization, supervision, software, validation, writing – original draft, and writing – review and editing.

## Conflicts of interest disclosure

The authors declare no conflicts of interest.

## Research registration unique identifying number (UIN)

Not applicable.

## Guarantor

Bao-Lin Qian.

## Data availability statement

The datasets used and/or analyzed in the current study are available from the corresponding author upon reasonable request.

## Provenance and peer review

This paper was not invited.
